# GABA_A_ Receptor α Subunits Differentially Contribute to Diazepam Tolerance after Chronic Treatment

**DOI:** 10.1371/journal.pone.0043054

**Published:** 2012-08-13

**Authors:** Christiaan H. Vinkers, Ruud van Oorschot, Elsebet Ø. Nielsen, James M. Cook, Henrik H. Hansen, Lucianne Groenink, Berend Olivier, Naheed R. Mirza

**Affiliations:** 1 Division of Pharmacology, Utrecht Institute for Pharmaceutical Sciences, Utrecht University, Utrecht, The Netherlands; 2 Department of Psychiatry, Rudolf Magnus Institute of Neuroscience, University Medical Center Utrecht, Utrecht, The Netherlands; 3 NeuroSearch A/S, NsDiscovery, Ballerup, Denmark; 4 Department of Chemistry and Biochemistry, University of Wisconsin-Milwaukee, Milwaukee, Wisconsin, United States of America; 5 Gubra ApS, Hørsholm, Denmark; 6 Department of Psychiatry, Yale University School of Medicine, New Haven, Connecticut, United States of America; 7 Aniona ApS, Ballerup, Denmark; Radboud University, The Netherlands

## Abstract

**Background:**

Within the GABA_A_-receptor field, two important questions are what molecular mechanisms underlie benzodiazepine tolerance, and whether tolerance can be ascribed to certain GABA_A_-receptor subtypes.

**Methods:**

We investigated tolerance to acute anxiolytic, hypothermic and sedative effects of diazepam in mice exposed for 28-days to non-selective/selective GABA_A_-receptor positive allosteric modulators: diazepam (non-selective), bretazenil (partial non-selective), zolpidem (α_1_ selective) and TPA023 (α_2/3_ selective). In-vivo binding studies with [^3^H]flumazenil confirmed compounds occupied CNS GABA_A_ receptors.

**Results:**

Chronic diazepam treatment resulted in tolerance to diazepam's acute anxiolytic, hypothermic and sedative effects. In mice treated chronically with bretazenil, tolerance to diazepam's anxiolytic and hypothermic, but not sedative, effects was seen. Chronic zolpidem treatment resulted in tolerance to diazepam's hypothermic effect, but partial anxiolytic tolerance and no sedative tolerance. Chronic TPA023 treatment did not result in tolerance to diazepam's hypothermic, anxiolytic or sedative effects.

**Conclusions:**

Our data indicate that: (i) GABA_A_-α_2_/α_3_ subtype selective drugs might not induce tolerance; (ii) in rodents quantitative and temporal variations in tolerance development occur dependent on the endpoint assessed, consistent with clinical experience with benzodiazepines (e.g., differential tolerance to antiepileptic and anxiolytic actions); (iii) tolerance to diazepam's sedative actions needs concomitant activation of GABA_A_-α_1_/GABA_A_-α_5_ receptors. Regarding mechanism, in-situ hybridization studies indicated no gross changes in expression levels of GABA_A_ α_1_, α_2_ or α_5_ subunit mRNA in hippocampus or cortex. Since selective chronic activation of either GABA_A_ α_2_, or α_3_ receptors does not engender tolerance development, subtype-selective GABA_A_ drugs might constitute a promising class of novel drugs.

## Introduction

Classical benzodiazepines allosterically enhance the inhibitory actions of GABA by binding to GABA_A_ receptors that contain α_1_, α_2_, α_3_ or α_5_ subunits in combination with a β and a γ_2_ subunit [1]. Despite widespread clinical utility, benzodiazepines are associated with tolerance development to some therapeutic effects over time. There is insufficient knowledge about the mechanisms involved in tolerance development to benzodiazepines nor why tolerance develops at different rates depending on the endpoint (sedation, anxiolytic, amnesic, anti-epileptic) [2], [3]. From a therapeutic perspective, this understanding is desirable as benzodiazepines are highly effective drugs which, if they could be prescribed for longer-term use, would offer major benefits to patients. In addition, tolerance mechanisms are also likely to be important in our understanding as to why a smaller group of patients escalate and abuse benzodiazepines. Although poorly understood, tolerance development has been ascribed to adaptation at the level of the GABA_A_ receptor, either as a consequence of changes in receptor expression or due to reduced allosteric coupling between GABA and benzodiazepine binding sites (see review by [2]. However, putative changes in GABA_A_ receptor subunit mRNA levels in response to chronic treatment are variable and suggested to be both treatment- and region-specific [4], [5].

Increased understanding that distinct GABA_A_ receptor subtypes mediate different effects of benzodiazepines has led to a new generation of α subunit-selective ligands, some of which have been tested in man [6]. Elegant genetic studies introduced single point mutations into specific α-subunits of the GABA_A_ receptor (α_1_(H101R), α_2_(H101R), α_3_(H126R) and α_5_(H105R)), making subunits insensitive to benzodiazepines without affecting GABA sensitivity [7]. The pharmacological approach has resulted in novel compounds which, whilst binding with equal affinity to all GABA_A_ receptor α subunits (α_1_, α_2_, α_3_ or α_5_), engender differential activation (intrinsic efficacy) of one or more of these receptors [6]. The availability of such subtype-selective tools allows one to test whether tolerance is mediated by the same GABA_A_ receptor subtype that are involved in the acute effect, or whether (i) specific α subunit receptors are involved in the development of tolerance, or (ii) a more complex interaction between α subunit receptors accounts for tolerance. However, despite the increased availability of subtype-selective pharmacological tools, understanding tolerance development to the different effects of benzodiazepines has received scant attention. One study has shown using α point-mutated mice that GABA_A_-α_5_ receptors are important in the development of tolerance to the sedative effects of diazepam [8]. Further, no analgesic tolerance was shown to the prototype α_2/3/5_ subtype-selective compound L838,417 (relative to morphine) [9] nor to the purported α_2/3_ selective ligand HZ166 [10] in a chronic constriction injury model of neuropathic pain.

Here, we compare tolerance to various acute effects of diazepam in rodents during chronic (28-day) treatment with the non-selective GABA_A_ receptor positive allosteric modulators (PAMs) diazepam (a full efficacy PAM) and bretazenil (a weak efficacy PAM), with the subtype selective GABA_A_-α_1_ affinity selective sedative-hypnotic PAM zolpidem [11], and the GABA_A_-α_2/3_ functionally selective PAM TPA023 [12]. Tolerance development to the anxiolytic and hypothermic effects of diazepam in these different treatment groups was assessed at 7-day intervals using the stress-induced hyperthermia (SIH) paradigm [13]. The SIH response is the transient rise in body temperature in response to a stressor that is mediated by the autonomic nervous system. The SIH response can be reduced using clinically effective anxiolytics including (subtype-selective) GABAergic compounds [14]. The SIH model was selected rather than another anxiolytic model, since the SIH response remains intact upon repeat testing at one week intervals [15], [16], whereas repeated exposure results in habituation in the light-dark box and the elevated plus maze [17], [18]. The primary aim of this study was to disentangle the role of different GABA_A_ receptor subtypes in tolerance development to the benzodiazepine diazepam, as well as to ascertain whether any molecular alterations after chronic treatment might account for tolerance development.

## Materials and Methods

### Animals

Male 129Sv/EvTac mice (Taconic, Denmark, 10–12 weeks old) were group-housed at constant temperature (21±2°C) and humidity (50%–60%) with PVC tubing as cage enrichment. Standard food pellets (Special Diet Services, Witham, Essex, United Kingdom) and water were freely available. Mice were maintained on a 12-hour light–dark cycle (lights on at 6 AM). One week after arrival, osmotic minipumps were implanted. After recovery from surgery, mice were group-housed. All experiments were approved by the Ethics Committee for Animal research at Utrecht University (2008.06.049) and in accordance with the declaration of Helsinki. All efforts were made to minimize the number of animals used and their suffering.

### Drugs

**Figure 1:**
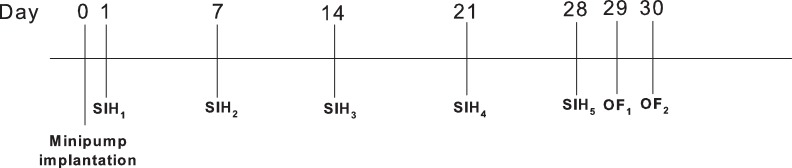
Chronological sequence of experimental studies/assessments in animals treated chronically with various GABA_A_ receptor positive allosteric modulators (see Methods and **Tables 2** & **3**). SIH: stress-induced hyperthermia test; OF: open field test. In experiment 2, only one OF was carried out.

Diazepam (base) and zolpidem (tartaric acid) were obtained from Sigma Aldrich. TPA023 (7-(1,1-dimethylethyl)-6-(2-ethyl-2H-1,2,4-triazol-3-ylmethoxy)-3-(2-fluorophenyl)-1,2,4-triazolo[4,3-b]pyridazine) was synthesized according to published methods [19]. Bretazenil was provided by Neurosearch A/S. An injection volume of 10 ml/kg was used for drug injections. A fresh diazepam suspension in gelatin-mannitol 0.5%/5% was prepared daily. A chronic vehicle control group was included in all experiments to control for the influence of repeat testing and to ascertain whether acutely administered diazepam itself led to tolerance. In the following methodological descriptions described below (post-surgery), we assessed the influence of chronic treatment with the GABAergic drugs described above on: (i) tolerance to diazepam's acute effects on SIH, hypothermia and locomotor activity (open field); and (ii) expression levels of various GABA_A_ α subunits using in-situ hybridisation. To aid in reading the following the methodology, and the subsequent results, Figure 1 should be referred to for the sequence of physiological, behavioural and molecular assessments made.

### Surgery

Mice were anaesthetized and osmotic minipumps (Type 2004, Alzet, USA) were subcutaneously implanted in the dorsal thoracic area delivering either vehicle or drug (in 95% PEG 400, 2.5% alcohol 70% and 2.5% distilled water; Sigma). Bretazenil was dissolved in 35% DMSO, 55% PEG-400 and 10% alcohol (70% v/v), identical with the vehicle. After experiments, minipumps were checked for the presence of any drug crystallization. Experimental procedures started the day after implantation (day 1).

### In vivo [^3^H]flumazenil Binding

*In vivo* binding studies with [^3^H]flumazenil were also conducted, to ascertain if drugs penetrated the mouse brain and occupied CNS benzodiazepine receptors after acute subcutaneous administration, allowing for valid inferences to be drawn. [^3^H]Flumazenil (79 Ci/mmol) was obtained from PerkinElmer Life and Analytical Sciences. The *in vivo* [^3^H]flumazenil binding studies were performed as reported previously by Atack et al [20]. Briefly, in two separate experiments, groups of female NMRI mice (25–28 g, n = 3/dose) were injected with [^3^H]flumazenil (2 µCi) via the tail-vein 20 minutes after receiving vehicle (saline), or four doses of diazepam (0.1–5 mg/kg), bretazenil (0.01–0.3 mg/kg), TPA023 (0.1–3 mg/kg), or zolpidem (0.3–10 mg/kg) s.c.”. Ten minutes later, mice were killed by decapitation, brains were rapidly removed and forebrains homogenized in 12 ml of ice-cold Tris citrate buffer (50 mM, pH 7.1) using a Ultra-Turrax homogenizer. Three separate aliquots of 1 ml were filtered through Whatman GF/C glass fiber filters and washed twice with 5 ml of ice-cold buffer. Nonspecific binding was determined by animal groups administered 3.0 mg/kg i.p. clonazepam 20 min before [^3^H]flumazenil injection. Radioactivity on the filters was determined by conventional liquid scintillation counting using a Tri-Carb™ counter (PerkinElmer). ED_50_ values were (Table 1) determined from dose-response curves based on the equation B = 100-(100·D/(ED_50_+D)), where B is the binding in percent of total specific binding; and D the dose of test compound.

**Table 1:** ED_50_ values for various GABA_A_ receptor positive allosteric modulators as derived from in-vivo binding of [^3^H]flumazenil to mouse forebrain.

Compound	ED_50_ (mg/kg)
Diazepam	3.9 (3.3–4.4)
Bretazenil	0.125 (0.12–0.13)
TPA023	0.22 (0.19–0.25)
Zolpidem	6.5 (5.5–7.5)

Data represent 2 independent experiments with n = 3 for all drugs in each experiment; range shown in parenthesis.

### Experiment 1: Tolerance Development to Diazepam in Mice Treated Chronically with Diazepam, Zolpidem or TPA023 (Table 2)

Mice (n = 10/group) were implanted with osmotic minipumps containing vehicle, diazepam, zolpidem or TPA023 (all groups received a dose of 15 mg/kg/day) (day 0). After surgery, seven animals died, leading to differences in group size across chronic treatment groups (Table 2). A higher chronic dose of all compounds including diazepam (15 mg/kg/day) was selected for this study compared to a previous experiment (Figure S1, and Table S1) due to only partial tolerance to diazepam's acute effects on SIH and hypothermia in animals receiving chronic diazepam at a dose of 10 mg/kg/day for 28 days (Figure S1). TPA023 and zolpidem were likewise administered at doses of 15 mg/kg/day (see Results section for further explanation for dose selection). Initially, SH-053-2F’-R-CH_3_ was included as a mixed affinity/functionally selective GABA_A_-α_5_ tool [21], [22], but subsequently excluded since it displayed no CNS displacement of [^3^H]flumazenil or [^3^H]L-655,708 after sc or iv administration (data available in Supplementary Information text). Exclusion of the SH-053-2F’-R-CH_3_ data did not result in changes to the overall results and interpretation. After the last testing day, mice that had received weekly vehicle test injections were decapitated, brains removed and stored at −80°C. Mice that had received weekly diazepam test injections were tested in the open field on days 29–30 (see below).

### Experiment 2: Tolerance Development to Diazepam in Mice Treated Chronically with Bretazenil (Table 3)

To ensure that low efficacy per se (as with TPA023, see [12]) did not have an impact on diazepam-induced tolerance regardless of selectivity, we tested the non-selective low efficacy PAM bretazenil [23]. Mice (n = 12/group) were implanted with osmotic minipumps containing either vehicle or the low efficacy PAM bretazenil (5 mg/kg/day). After surgery, three animals died, leading to differences in group sizes across chronic treatment groups (Table 3). The dose of bretazenil (5 mg/kg) was lower compared to other drugs used in Experiment 1 because dissolution of bretazenil proved to be difficult at higher concentrations. On day 29, all mice were tested in the open field (see below).

### Behavioural Procedures

#### Stress-induced hyperthermia (SIH) procedure

The SIH procedure was carried out according to methodology published previously [24]. SIH is quantified in singly housed mice using rectal temperature assessment as the stressor. Rectal temperature is measured twice 10-min apart, with the first measurement serving as the stressor and the second as a measure of the stress response. Due to the stress experienced during the first temperature (*T*_1_) measurement, the temperature of the second measurement (*T*_2_) is approximately 1–2°C higher than that of the first (*T*_1_). This difference in temperature (Δ*T* = *T*_2_−*T*_1_) is defined as the SIH response. In determining diazepam's influence on the SIH response in the different chronic treatment groups outlined in Experiments 1 and 2 described above, either vehicle or diazepam (5 mg/kg, IP) was injected 60 min before the first temperature measurement (T_1_). Methodological experiments have shown that an injection interval of 60 min or longer prior to the first temperature measurement results in a hyperthermic response comparable to animals not injected at all [15]. Also, previously it has been shown that using a 60-min injection-stress interval, injections with different vehicle solutions resulted in an identical SIH response compared to animals that had not received any injection [13] Drug administration was pseudorandomized to ensure that comparable drug challenges occurred in the morning and afternoon. Body temperature was measured by inserting a silicon oil dipped thermistor probe up to 2 cm in to the rectum for 15–20s.

**Table 2:** Experimental protocol of repeat weekly testing with acute administration of vehicle or diazepam (5 mg/kg, IP) in mice chronically administered vehicle, diazepam, zolpidem or TPA023 (15 mg/kg/day) for 4-weeks via subcutaneously implanted osmotic minipumps (**Experiment 1**).

Experiment 1		
Chronic (N)	Selectivity profile	Acute Testing (day 1-7-14-21-28)
Vehicle (10)	–	Vehicle
Vehicle (10)	–	Diazepam
Diazepam (7)	α_1/2/3/5_	Vehicle
Diazepam (8)	α_1/2/3/5_	Diazepam
Zolpidem (9)	α_1_	Vehicle
Zolpidem (9)	α_1_	Diazepam
TPA023 (10)	α_2/3_	Vehicle
TPA023 (10)	α_2/3_	Diazepam

**Table 3:** Experimental protocol of repeated weekly testing with acute administration of vehicle or diazepam (5 mg/kg, IP) in mice chronically administered vehicle or bretazenil (5 mg/kg/day) for 4-weeks via subcutaneously implanted osmotic minipumps (**Experiment 2**).

Experiment 2		
Chronic (N)	Selectivity profile	Acute Testing (day 1-7-14-21-28)
Vehicle (11)	–	Vehicle
Vehicle (12)	–	Diazepam
Bretazenil (10)	α_1/2/3/5_	Vehicle
Bretazenil (12)	α_1/2/3/5_	Diazepam

#### Open field test

The influence of the various chronic treatments, described in Experiments 1 and 2 above, on diazepam-induced hypolocomotion was assessed using an open field (Ø 20 cm). In Experiment 1, mice that had been tested weekly with acute diazepam were tested for tolerance to diazepam's hypolocomotor effect at the end of the 28-day treatment period using a within-subject design. In Experiment 2, all mice were tested in the open field using a between-subject design. In both experiments, diazepam (5 mg/kg, IP) or vehicle was administered 60 min prior to testing in the open field. Each animal was placed in the dimly lit center (5 lux) and allowed to explore for 5 min. The distance travelled was determined using an automatic tracking system (TSE ActiMot V7.01, TSE systems GmbH, Bad Homburg, Germany).

#### In-situ hybridization

Mice from Experiment 1 that had received weekly acute vehicle injection challenges were used to determine if there was any influence of the various chronic treatments (vehicle, diazepam, bretazenil or TPA023) on the expression of α_1_, α_2_, α_3_, α_5_ subunit mRNA in the hippocampus. Moreover, in the cortex specifically assessed the influence of chronic diazepam treatment only on the expression of α_1_, α_2_, α_3_, α_5_ subunit mRNA. Mice that had received weekly acute diazepam challenges were not used since we were concerned that even these brief weekly challenges with diazepam may have influenced expression levels GABA_A_ α subunits. For the hippocampal CA1 region, the *n* sizes were as follows: α_1_(n = 7–11); α_2_ (n = 7–11); α_5_ (n = 6–10), and for the cortex: α_1_ (n = 6); α_2_ (n = 6–7); α_5_ (n = 5–6).Twelve micrometer thick serial coronal brain cryostat sections were cut through the cortex and dorsal hippocampus (1.94 mm posterior to bregma [25]. For each region, one glass slide with at least three brain sections from each animal were processed together to achieve maximal standardization. Slides were fixed for 5 min in 4% paraformaldehyde in 0.2 M PBS, acetylated and finally delipidated and dehydrated as described earlier [26]. Synthetic oligonucleotide DNA probes (DNA Technology, Aarhus, Denmark) complimentary to the mouse GABA_A_ α_1_ (GTTGTTTTTCTTAATGAGAGGATCCTTTACTTTCTTTGGCTTTTCTGG, targeting bases 1507–1554, acc. no. BC137733), α_2_ (GAGGACAGGATCTTTGGAAAGATTCGGGGCATAGTTGGCAACAGCTAC, targeting bases 1318–1365, acc. no. BC115727), α_3_ (TGAAGCAATCGCTGTTGGAGTTGAAGAAGCACTGGGAGCAGCAGCAGA, targeting bases 1327–1372, acc. no. BC119317) and α_5_ (GCATTCGCGGTCCCAGCTGGAGGCTGCTCTTTTGGGATGTTTGGAGGA, targeting bases 1497–1544, acc. no. BC062112) subunits were used. The oligonucleotide probes were labelled at the 3'-end with [^35^S]dATP (3000 Ci/mmol, GE Healthcare, UK) using terminal deoxynucleotidyl transferase (Roche Diagnostics GmbH, Germany) at a specific activity of 1x10^6^ cpm/100µl in the hybridization buffer. Sections were hybridized and washed as described previously [26], and exposed together with [^14^C] standards to a Kodak BiomaxMR (GE Healthcare) film for 2–3 weeks and scanned at the highest resolution (GS-800 scanner, Bio-Rad, Hercules, CA). Optical densities from the autoradiograms were quantified in the retrosplenial/primary motor cortex and the hippocampal CA1 region using an image analysis program (Quantity-One software v.4.6, Bio-Rad, Hercules, CA) by a person blind to the allocated treatment. The individual value for each animal was the average of three individual sections measured bilaterally within the area of interest. Background values were measured for each slide individually in an area without tissue.

### Data Analysis

#### General

A probability level of *p*<0.05 was set as statistically significant for all datasets. All data are displayed as mean ± S.E.M. Dunnett’s post-hoc test was used throughout, unless otherwise indicated.

#### Stress-induced hyperthermia and body temperature

Treatment effects on the SIH response (SIH response = T_2_–T_1_) and basal body temperature (T_1_) were evaluated using a repeated-measures analysis of variance (ANOVA) with time (weeks) as a within-subject, and acute treatment and chronic treatment as between-subject factors.

#### Open field test

For Experiment 1, the open field test was analyzed using repeated-measures ANOVA with acute treatment (diazepam/vehicle) as a within-subject factor and chronic treatment (diazepam/zolpidem/TPA023/vehicle) as between-subject factor. In Experiment 2, analysis was carried out using a two-way ANOVA with acute treatment (diazepam/vehicle) and chronic treatment (bretazenil/vehicle) as between-subject factors.

#### In situ hybridization

Chronic treatment effects on GABA_A_ receptor subunit expression levels were evaluated using a one-way ANOVA with Tukey's post-hoc test (hippocampal CA1 region) or an unpaired two-tailed t-test (cortex).

## Results

### In vivo [^3^H]flumazenil Binding to Mouse Forebrain

In mice administered diazepam, bretazenil, TPA023 and zolpidem there was a dose-dependent displacement of [^3^H]flumazenil from mouse forebrain 30 minutes after s.c. dosing. The ED_50_'s for displacement were: diazepam 3.9 mg/kg, bretazenil 0.125 mg/kg, TPA023 0.22 mg/kg, and zolpidem 6.5 mg/kg (Table 1). ED_50_ values were comparable to published data [27]. Although these studies were conducted after acute s.c. administration of compounds, the data indicate that sufficient CNS penetration can be achieved after (chronic) peripheral administration.

### Experiment 1: Tolerance Development to Diazepam in Mice Treated Chronically with Diazepam, Zolpidem or TPA023 (Table 2)

#### SIH response (Figure 2)

**Figure 2:**
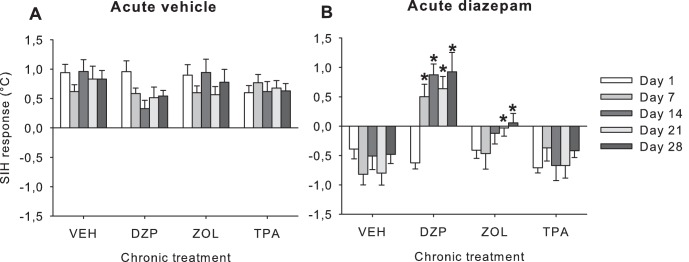
Effect of acute vehicle or diazepam (5 mg/kg, IP) administration on the stress-induced hyperthermia (SIH) response measured 60 minutes post-administration in mice treated chronically with various different GABA_A_ receptor positive allosteric modulators (see Methods and **Table 2** **).** *: p<0.05 acute diazepam effect compared to day 1. VEH: vehicle; DZP: diazepam; ZOL: zolpidem; TPA: TPA023.

Tolerance to diazepam's acute effects on the SIH response was dependent upon which chronic treatment mice had received (chronic x acute treatment interaction F_3,65_ = 10.47,p<0.001), and was influenced by time (time x chronic x acute treatment interaction F_12,260_ = 3.26,p<0.001, Figure 2B). Post hoc analysis showed that, compared to chronic vehicle treatment, in mice treated chronically with diazepam there was a loss of acute diazepam's effect on SIH starting from day 7 (day 7: p<0.001; day 14: p<0.05; day 21: p<0.001; day 28: p<0.01), whereas in chronic zolpidem-treated mice, tolerance development to diazepam's acute SIH-reducing effects did not occur until day 21 (day 21: p<0.05; day 28: p<0.05). By contrast, a consistent diazepam-induced reduction of the SIH response was demonstrable in mice treated chronically with TPA023 throughout the 28 days. This shows that there was no significant loss in diazepam’s acute effects in the TPA023 treated group compared to the chronic vehicle treated group.

In the parallel control study there was no significant effect of weekly acute vehicle challenge injections nor a significant effect of chronic treatment itself on the SIH response in mice treated chronically with vehicle, diazepam, zolpidem or TPA023 (Figure 2A).

#### Basal body temperature (Figure 3)

**Figure 3:**
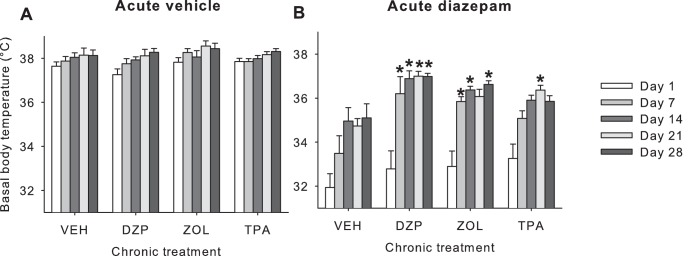
Effect of acute vehicle or diazepam (5 mg/kg, IP) administration on basal body temperature measured 60 minutes post-administration in mice treated chronically with various different GABA_A_ receptor positive allosteric modulators (see Methods and **Table 2** **).** *: p<0.05 compared to the acute diazepam on day 1. VEH: vehicle; DZP: diazepam; ZOL: zolpidem; TPA: TPA023.

Overall, all chronic treatments led to a loss of diazepam-induced hypothermia (chronic x acute treatment interaction F_3,65_ = 6.24,p = 0.001) independent of testing day (time x chronic x acute treatment interaction p = 0.48, compare Figure 3B with Figure 3A). Post hoc analysis indicated that acute diazepam challenge led to a greater loss of diazepam-induced hypothermia in chronic diazepam and zolpidem treated animals compared to chronic vehicle-treated mice across days 7–28 (P<0.05). However, a significant loss of diazepam induced hypothermia in the Chronic TPA023 treated group was only demonstrable day 21 (p<0.05). In the parallel control study there was no significant effect of acute vehicle treatment nor a significant effect of chronic treatment itself on basal body temperature in mice treated chronically with vehicle, diazepam, zolpidem or TPA023 (Figure 3A).

#### Open field test (Figure 4)

Chronic treatment with different GABA_A_ PAMs differentially affected diazepam-induced hypolocomotion in the open field (total distance travelled: acute x chronic interaction F_3,31_ = 4.47,p<0.01; percentage of time moving: acute x chronic interaction F_3,31_ = 4.53,p = 0.01). Post hoc analysis indicated that acute diazepam injection reduced locomotor activity (both the total distance travelled and the percentage of time moving) in animals that had been chronically treated with vehicle (p<0.001), zolpidem (p<0.01), or TPA023 treatment (p<0.01) - but not in animals that had been chronically treated with diazepam (total distance travelled: p = 0.88; percentage of time moving: p = 0.67), indicating tolerance only in chronic diazepam treated mice.

**Figure 4:**
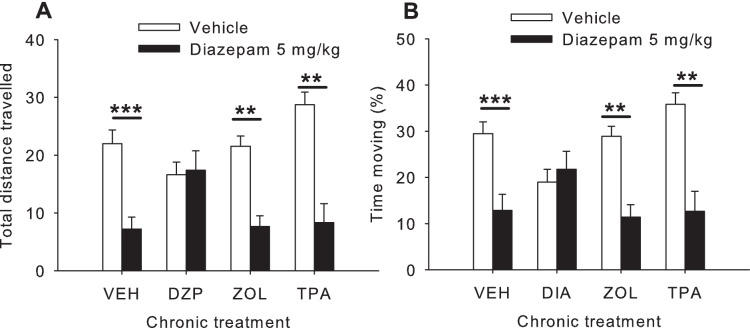
Effect of acute diazepam (5 mg/kg IP) or vehicle administration on total distance travelled (A) and percentage of time moving (B) in the open field, in mice treated chronically with various different GABA_A_ receptor (see Methods and **Table 2** **) for approximately 4 weeks (Experiment 2, see text).** **: p<0.01 compared to vehicle. VEH: vehicle; DZP: diazepam; ZOL: zolpidem; TPA: TPA023.

#### In situ hybridization (Figure 5)

The mouse hippocampus exhibited high levels of GABA_A_ receptor α_1_, α_2_, and α_5_ subunit mRNAs. The almost undetectable levels of α_3_ subunit mRNA expression in the hippocampus precluded consistent quantification of α_3_ subunit mRNA. At this coronal level, moderate expression of the α_1_ subunit transcript was also detected in the cerebral cortex, mediodorsal, ventral thalamus, and hypothalamus. While the hippocampus was almost devoid of α_3_ expression, exhibiting only very sparse representation in the CA1 region, the cortex, laterodorsal thalamus and hypothalamus showed moderate expression (data not shown). The α_5_ subunit was also expressed to a modest degree in the cortex as well as in the hypothalamus. The distribution patterns of α_1_, α_2_, α_3_ and α_5_ subunit mRNAs is in general agreement with previous reports [28], [29]. A one-way ANOVA showed that there was an effect of chronic treatment with different GABA_A_ PAMs on the level of mRNA for the α_2_ subunit in the CA1 (p<0.05). Specifically in animals receiving chronic diazepam treatment there was a significant increase in α_2_ mRNA expression level. By contrast, there was no influence of chronic diazepam treatment on α_2_ mRNA expression level in the cortex nor any other significant effect of chronic treatments on α_1_ or α_5_ mRNA expression level in the hippocampus CA1 region or the cortex (p>0.05 in all cases).

**Figure 5:**
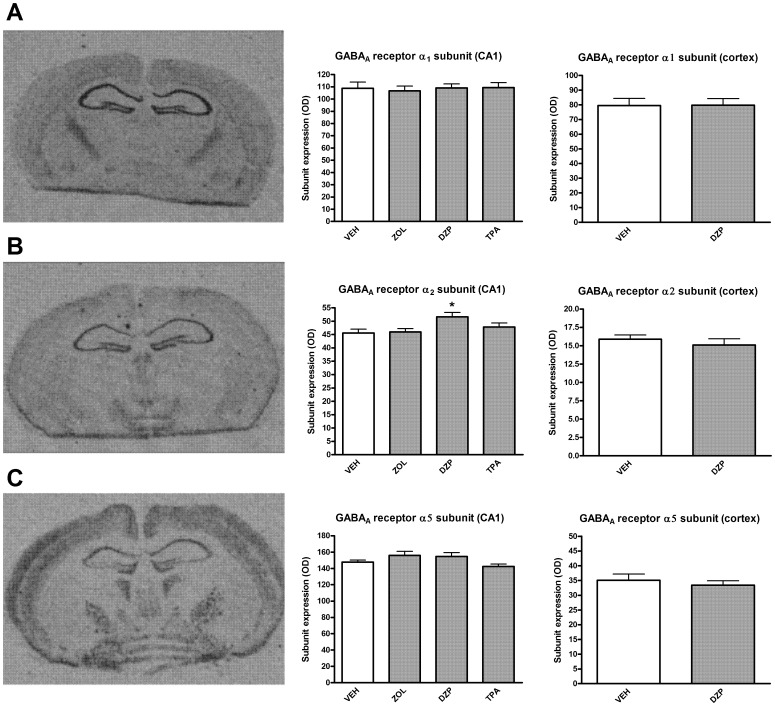
Differences in expression of GABA_A_ receptor α_1_ (A), α_2_ (B) and α_5_ (C)mRNA in the hippocampus and cortex (retrosplenial and primary motor cortex) in mice chronically treated with various GABA_A_ receptor (see Methods and **Table 2**). *: p<0.05.

### Experiment 2: Tolerance Development to Diazepam in Mice Treated Chronically with Bretazenil (Table 3)

#### SIH response (Figure 6A)

Chronic bretazenil treatment led to tolerance to diazepam’s SIH reducing effects (acute x chronic treatment interaction F_1,41_ = 7.19,p<0.01). Post hoc analysis of the separate testing days showed that on days 7 and 28, acute diazepam treatment had a significantly curtailed SIH-reducing effect in mice chronically treated with bretazenil compared to chronic vehicle treated mice (day 1 (p = 0.61); day 7 (p<0.05); day 14 (p = 0.10); day 21 (p = 0.12) and day 28 (p<0.05). In separate analysis of the chronic vehicle treated mice, diazepam reduced the SIH response regardless of testing week (diazepam effect F_1,21_ = 72.21,p<0.001; time x diazepam interaction p = 0.22). In contrast, the efficacy of diazepam changed over time in mice chronically treated with bretazenil (time x diazepam interaction F_4,80_ = 2.94,p<0.05).

**Figure 6:**
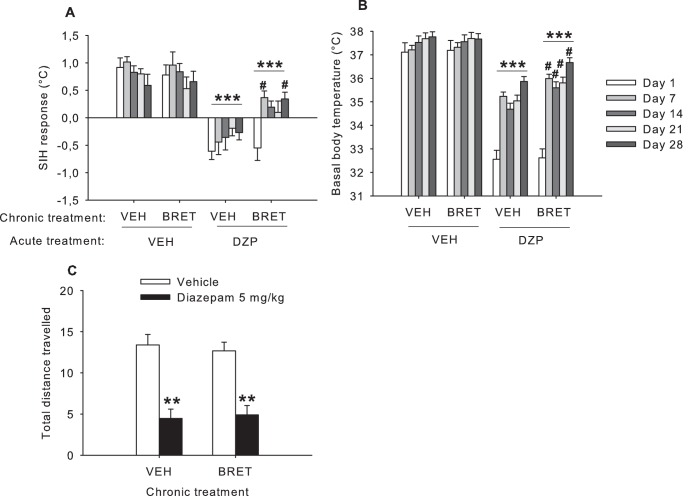
Effect of acutely administered vehicle or diazepam (5 mg/kg, IP) on (A) the stress-induced hyperthermia response, (B) basal body temperature and (C) distance travelled in the open field measured 60 minutes post-administration, in mice treated chronically with bretazenil (5 mg/kg/day) or vehicle (Experiment 3). ***: p<0.001 (**: p<0.01) compared to acute vehicle administration. #: p<0.05 compared to the chronic vehicle group acutely receiving diazepam. BRET: bretazenil; DZP: diazepam; VEH: vehicle.

#### Basal body temperature (Figure 6B)

Diazepam-induced hypothermia was dependent upon the chronic treatment mice had received (acute x chronic treatment F_1,41_ = 3.46,p = 0.05). There was significant tolerance to acute diazepam-induced hypothermia in chronic bretazenil-treated compared to chronical vehicle-treated mice (chronic treatment effect F_1,22_ = 5.52,p<0.05). By contrast, after acute vehicle administration, there were no chronic treatment effects (chronic treatment effect F_1,19_ = 0.03,p = 0.87, NS; time x chronic treatment interaction p = 0,98). Further post hoc analysis show that, compared to chronic vehicle treatment, significant tolerance to diazepam's hypothermic effects was demonstrable in the chronic bretazenil treatment group on days 7, 14, 21 and 28 (p<0.05).

#### Open field test (Figure 6C)

Chronic treatment with bretazenil did not affect diazepam-induced hypolocomotion in the open field test (total distance traveled: diazepam effect F_1,41_ = 33.96. p<0.001; chronic x acute treatment interaction p = 0.49; percentage of time moving: diazepam effect F_1,41_ = 23.42. p<0.001; chronic x acute treatment interaction p = 0.68).

## Discussion

In the present study, we investigated whether differential tolerance develops to the anxiolytic-like, hypothermic and sedative effects of acute diazepam administration in mice treated chronically for 28 days with various GABA_A_ receptor PAMs with different affinity and/or functional selectivity for GABA_A_ receptor subtypes [6], [30]. Tolerance development appears to have different quantitative and temporal dynamics dependent on the endpoint assessed (*e.g.*, anxiety, hypothermia or hypolocomotion) [3]. This is broadly in line with the clinical situation where differential tolerance to the effects of benzodiazepines exists dependent upon the clinical endpoint. Probably, these different "tolerance profiles" in rodents or in humans are a consequence of multiple factors including dose, exposure, receptor occupancy, half-life, metabolic and pharmacokinetic profiles, in addition to affinity, efficacy and subtype selectivity [27]. One clinical implication of our data is that GABA_A_-α_2_/α_3_ subtype selective ligands such as TPA023 are unlikely to lead to anxiolytic tolerance. However, to date there is still no firm evidence that GABA_A_-α_2_/α_3_ subtype selective compounds are anxiolytic in man [30].

In two separate studies, we demonstrated that during chronic 28-day treatment with the classical non-selective benzodiazepine diazepam (10 or 15 mg/kg), there was tolerance to acute diazepam's anxiolytic and hypothermic effects. Interestingly, in the first experiment where diazepam was administered via osmotic minipumps at a dose of 10 mg/kg there was partial and slowly developing anxiolytic tolerance (by day 21), whereas there was relatively robust and rapid hypothermic tolerance (by day 7–14) (see Figure S1 and Table S1). By contrast, in the follow up experiment where a higher dose (15 mg/kg) of diazepam was administered for 28 days, a more robust anxiolytic tolerance was present (Figure 2). The rate of tolerance development to the anxiolytic and hypothermic effects after acute diazepam administration were comparable across both experiments. Clearly, the dose level of diazepam has a greater impact on the rate and level of anxiolytic tolerance development compared to hypothermic tolerance. Extrapolation of this mouse dose to a human equivalent is performed through normalization to body surface area [31]. This way, an acute diazepam dose of 5 mg/kg in mice corresponds to an equivalent human dose of 0.40 mg/kg.

To understand whether tolerance to effects of diazepam described above could be ascribed to specific subtypes of GABA_A_ receptors, we ran additional studies by assessing acute diazepam challenges in animals chronically treated via osmotic mini-pumps with different subtype selective PAMs. However, since some recently described PAMs with functional selectivity for subtypes of GABA_A_ receptors are not full efficacy PAMs (*i.e.*, partial agonists) at receptors for which they possess functional selectivity [32], we tested the non-selective low efficacy PAM bretazenil to ensure low efficacy per se did not have an impact on diazepam-induced tolerance independent of selectivity. In animals chronically treated with the low-efficacy PAM bretazenil, tolerance to diazepam's anxiolytic and hypothermic effects was broadly consistent with tolerance in chronic diazepam treated mice. However, the magnitude of anxiolytic tolerance was somewhat lower in chronic bretazenil-treated mice than chronic diazepam-treated mice, consistent with a lower propensity to tolerance development with weak PAMs [23].

After chronic treatment with the α_1_-selective drug zolpidem [11], tolerance also developed to diazepam's acute anxiolytic and hypothermic effects. However, whereas the rate and level of tolerance development to diazepam-induced *hypothermia* was equivalent in chronic diazepam and zolpidem treated mice, the level and rate of *anxiolytic* tolerance were reduced in chronic zolpidem-treated animals (Figure 2). Since, (i) the anxiolytic effect of benzodiazepines such as diazepam are thought to be mediated via GABA_A_- α_2_ and/or α_3_ receptors, and (ii) zolpidem has 10–15 fold affinity selectivity for GABA_A_-α_1_ over GABA_A_-α_2_/α_3_ receptors, and no affinity for GABA_A_-α_5_ receptors [33], the partial anxiolytic tolerance to acute diazepam challenge in chronic zolpidem treated mice might imply: (a) that activation of GABA_A_-α_2_/α_3_ receptors does not lead to anxiolytic tolerance, or (b) that, given zolpidem's affinity selectivity profile, there was insufficient occupancy and activation of GABA_A_-α_2_/α_3_ receptors at the dose of zolpidem used to demonstrate anxiolytic tolerance to acute diazepam challenge. Our data with the α_2/3_ selective PAM TPA023 described below indicates that the former is more likely to be the case.

Conversely, since zolpidem has a >1000-fold affinity selectivity for GABA_A_-α_1_ over GABA_A_-α_5_ receptors, it would appear that the equivalent rate and level of hypothermic tolerance to acute diazepam challenge in chronic zolpidem and diazepam treated mice indicates that hypothermia is probably mediated through GABA_A_-α_1_ receptors and that there is rapid tolerance to this effect. These conclusions are consistent with our previous finding that the hypothermic response to zolpidem and diazepam in mice is antagonized by the GABA_A_-α_1_ selective antagonist β-CCT, whereas this same antagonist does not affect the SIH reducing effect of these two ligands [14]. However, a role for GABA_A_-α_2_/α_3_ receptors in mediating hypothermic tolerance to acute diazepam challenge cannot be fully excluded since zolpidem is only 10–15 fold affinity selective for GABA_A_-α_1_ over GABA_A_-α_2_/α_3_ receptors [1], and at the dose used here this might be sufficient to activate these receptor subtypes in-vivo. Also, we cannot fully exclude the possibility that the presence of alcohol in pumps may have potentially confounded our data, even though the amounts are well marginal compared to the acute doses of 2–4 g/kg that are needed to affect body temperature [34].

In contrast to anxiolytic tolerance to acute diazepam in chronic diazepam and, partly, in zolpidem treated mice, no anxiolytic tolerance developed to acute diazepam challenge in mice treated chronically with TPA023. Since TPA023 is functionally selective for GABA_A_-α_2_/α_3_ receptors which mediate benzodiazepine-induced anxiolysis [12], this data would appear to more directly support the contention that chronic activation of GABA_A_-α_2_/α_3_ receptors does not lead to anxiolytic tolerance to acute diazepam challenge. This conclusion would also concur with the fact that TPA023 does not activate GABA_A_-α_1_/α_5_ receptors despite high affinity binding to these receptor subtypes, and is consistent with the zolpidem data discussed above. Although a different endpoint, recent data have indicated no analgesic tolerance to the prototype α_2/3/5_ subtype-selective compound L838,417 (relative to morphine) [9] and the purported α_2/3_ subtype-selective ligand HZ166 [10] in a chronic constriction injury model of neuropathic pain.

Clinically, benzodiazepine tolerance to the anxiolytic effects of diazepam develops slowly or not at all [35], and our data suggest that chronic treatment with compounds selective for GABA_A_-α_2_/α_3_ receptors would be even less likely to induce tolerance to anxiolytic effects in man. Nonetheless, we would advice some caution in reaching this conclusion for two reasons: (i) it is only based on the autonomic SIH paradigm which may not be readily extrapolated to other anxiety paradigms like the elevated plus maze and the light-dark box. Although, in contrast to these paradigms [17], [18], a clear advantage of the SIH paradigm is that weekly use does not lead to adaptation of the unconditioned SIH response, and therefore, we were able to follow tolerance development over time without the confounds of repeat testing which are troublesome in other behavioural models of anxiety [36]; (ii) it is not clear what concentration of TPA023 is achieved after a 15 mg/kg dose in-vivo, specifically in the CNS. If the concentrations achieved are in excess of concentrations assessed in electrophysiology at recombinant GABA_A_ receptors [12], it is plausible that the *in-vivo* selectivity of the compound could differ from its *in vitro* selectivity profile. Certainly at the dose of TPA023 used (15 mg/kg) we would expect full saturation of central benzodiazepine sites on GABA_A_ receptors based on our in-vivo [^3^H]flumazenil binding data.

With respect to the hypothermia endpoint, although in mice treated chronically with TPA023 there appeared to be some hypothermic tolerance, this partial tolerance was also seen in chronic vehicle treated mice that had been challenged with acute diazepam in both experiments, suggesting no specific role for GABA_A_-α_2/_α_3_ receptors in hypothermia (Figure 3B). Finally, it is important to note that some degree of hypothermic tolerance, but not anxiolytic tolerance, developed after repeated intermittent diazepam challenge in chronic vehicle treated mice. This is relevant since intermittent treatment in animal studies is likely to be more comparable to intermittent exposure to drug in the clinical context rather than constant delivery of drug via osmotic minipumps. The constant and slow release of diazepam via the osmotic minipump may also explain why chronic diazepam treatment – in contrast to acute diazepam injections via the intraperitoneal route – did not result in any hypothermia (Figure 3A).

One additional observation to these main points regarding tolerance to the hypothermic tolerance to acute diazepam challenge, was that on day 1 acute diazepam challenge induced a rather severe hypothermic response in all chronic treatment groups compared to what we have demonstrated previously with this dose of diazepam [37]. It is likely that this severe hypothermic response is the result of a combination of both acute diazepam challenge and limited postsurgical recovery time (studies started one day after surgery). This severe hypothermia likely explains the death of seven animal's postsurgically (see Methods section), especially in the group that received diazepam via osmotic minipumps combined with an acute diazepam challenge on day 1. Although group sizes were still sufficient to allow for valid statistical analyses, in light of this severe hypothermic response to diazepam on day 1 in all groups, it is reasonable expressing some caution in interpreting the SIH results described above since hypothermia would interfere with thermoregulatory homeostatic processes essential to the SIH response. Moreover, since we did not include a no-stress group, we cannot completely exclude the possibility that recovery of diazepam-induced hypothermia itself could lead to an increase in body temperature independent of stress.

Interestingly, mice chronically treated with diazepam for just over 4 weeks displayed clear tolerance to acute diazepam-induced hypolocomotion. Tolerance to this effect of diazepam was not seen in any other chronic treatment group, including chronic bretazenil-treated mice. The bretazenil data are consistent with prior research indicating a lower propensity to tolerance development with weak PAMs [23] (but see also [38], [39]). Since motor deficits such as hypolocomotion and ataxia are mediated via GABA_A_-α_1_ receptors [40], [41], it is at first glance surprising that there is no tolerance to diazepam-induced hypolocomotion in chronic zolpidem treated mice since zolpidem has selectivity for the GABA_A_-α_1_ receptor [33]. However, tolerance to diazepam-induced hypolocomotion appears to depend on concomitant activation of GABA_A_-α_5_ receptors in addition to GABA_A_-α_1_ receptors [8]. These authors showed that sedative tolerance to 8-day diazepam treatment (15 mg/kg, IP) was observed in wildtype and GABA_A_- α_2_ and α_3_ knock-in mice, but not in GABA_A_-α_5_ knock-in mice. In the same study, binding experiments with the GABA_A_-α_5_ selective radioligands [^3^H]RY80/[^3^H]L655,708 showed that there was a significant decrease in binding in the dentate gyrus, concomitant with tolerance development, in wildtype and GABA_A_-α_2_ knock-in mice, but not GABA_A_-α_1_ knock-in mice. These findings are consistent with our findings here that there is no tolerance to diazepam-induced hypolocomotion in chronic zolpidem-treated mice, since zolpidem does not have affinity for the GABA_A_-α_5_ receptor [33]. Also, chronic zolpidem treatment in rodents fails to alter α_5_ subunit mRNA levels [42]. TPA023, whilst having high nM affinity for both GABA_A_-α_1_ and α_5_ receptors does not activate either of these receptor subtypes [12] and therefore cannot engender relevant (unknown) regulatory changes necessary for tolerance to diazepam-induced hypolocomotion.

Regarding molecular mechanisms underlying some of the physiological and behavioural tolerance data described above, our findings on the effect of chronic treatment with selective and non-selective PAMs on α_1_, α_2_ or α_5_ subunit mRNA levels in hippocampus and cortex give no obvious answers. Apart from the modest increase in hippocampal α_2_ subunit mRNA following chronic diazepam treatment, none of the other chronic drug treatments led to any significant changes in expression levels of α_1_, α_2_ or α_5_ subunit mRNA in hippocampus or cerebral cortex (Figure 5). Thus, overall no obvious alteration in GABA_A_ receptor subunit levels can account for tolerance development [35]. A recent extensive review yielded inconsistent changes of GABA_A_ receptor subunit mRNA and protein levels after chronic benzodiazepine treatment [5]. For example, there are studies that report no changes in α_1_ subunit mRNA expression after chronic benzodiazepine treatment [43], [44], [45], whereas others report decreases [46], [47], [48], [49]. Similar heterogeneity of results exists in reports on other GABA_A_ receptor subunits [50], [51], [52], [53], [54]. Putative changes in GABA_A_ receptor subunit mRNA levels appear to be both treatment- and region-specific, and may also depend on the method used to quantify mRNA levels. Moreover, the length and method of chronic treatment seems relevant, since differences in GABA_A_ receptor subunit mRNA levels after chronic diazepam treatment in rats can depend on whether diazepam is administered as daily systemic injections or via osmotic minipumps [55]. In addition to suggestions that tolerance development might reflect reduced allosteric interaction between the benzodiazepine and GABA binding sites [56], [57], [58], future studies on compensatory changes distal to the GABA_A_ receptor after chronic treatment with GABA_A_ receptor PAMs, including second messenger systems (e.g., [59]) or changes in excitatory amino acid systems [60], might shed more light on mechanisms underlying tolerance development.

In summary, our findings are a first step towards addressing the issue of tolerance development to chronic treatment with novel subtype selective GABA_A_ receptor PAMs. Broadly, our finding that tolerance development to diazepam in rodents has different quantitative and temporal dynamics dependent on the endpoint assessed is reminiscent of benzodiazepine effects in humans. This gives weight to rodents as a model system to study pharmacology and mechanisms associated with chronic benzodiazepine treatment. More specifically, our data with TPA023, relative to data with the other PAMs studied, leads us to conclude that tolerance development to GABA_A_-α_2_/α_3_ subtype selective drugs might not be an issue in man (see also [9], [10]). One clinical inference that might be drawn from our data is that a patient with an anxiety disorder treated with a GABA_A_-α_2_/α_3_ subtype selective drug is still likely to respond to a benzodiazepine, albeit to date there is still no firm evidence that GABA_A_-α_2_/α_3_ subtype selective compounds are anxiolytic in man [30]. However, our data does not address the converse clinical situation. It remains to be investigated whether a patient would continue to benefit from anxiolytic effects if switched from a classical benzodiazepine to a GABA_A_-α_2_/α_3_ subtype selective drug. It is important for future research to investigate whether anxiolytic cross-tolerance to the effect of TPA023 (or other subtype selective compound) is demonstrable in rodents chronically exposed to diazepam. If GABA_A_-α_2_/α_3_ subtype selective drugs do not induce tolerance in man, such subtype selective compounds might not engender clinical dependence [12], [61] or potentially drug abuse [62], [63]. This would constitute a significant clinical improvement over classical benzodiazepines in addition to GABA_A_-α_2_/α_3_ selective compounds' reduced propensity to engender sedation and postural instability [64]. In conclusion, this first study to systematically address tolerance development to GABA_A_-α_2_/α_3_ subtype-selective tools is an important step in improving our understanding of these novel pharmacological agents which might constitute a promising future drug class.

## Supporting Information

Figure S1 **Effect of acutely administered vehicle or diazepam (5 mg/kg, IP) on the stress-induced hyperthermia response (A) and basal body temperature (B) measured 60 minutes post-administration, in mice treated chronically with diazepam (10 mg/kg/day) or vehicle.** ***: p<0.001 (**: p<0.01) compared to acute vehicle administration. #: p<0.05 compared to acute diazepam administration in the chronic vehicle group. DZP: diazepam; Veh: vehicle.
(EPS)

Table S1 **Experimental protocol of repeated weekly testing with acute administration of vehicle or diazepam (5 mg/kg, IP) in mice chronically administered vehicle or diazepam (10 mg/kg/day, IP) for 4-weeks via subcutaneously implanted osmotic minipumps.**
(DOC)
