# Supplementary material for: GABAA Receptor α Subunits Differentially Contribute to Diazepam Tolerance after Chronic Treatment
Source: PLoS One. 2012 Aug 13;7(8):e43054. doi: 10.1371/journal.pone.0043054 (PMC3418228; doi:10.1371/journal.pone.0043054)
Supplement: Table S1 — Experimental protocol of repeated weekly testing with acute administration of vehicle or diazepam (5 mg/kg, IP) in mice chronically administered vehicle or diazepam (10 mg/kg/day, IP) for 4-weeks via subcutaneously implanted osmotic minipumps. (DOC) [file pone.0043054.s002.doc]

| **Chronic** | **Acute Testing (day 1-7-14-21-28)** |
| --- | --- |
| Vehicle (N=10) | Vehicle |
| Vehicle (N=10) | Diazepam |
| Diazepam (N=10) | Vehicle |
| Diazepam (N=10) | Diazepam |
